# Retraction: Xianglian pill suppresses inflammation and protects intestinal epithelial barrier by promoting autophagy in DSS induced ulcerative colitis mice

**DOI:** 10.3389/fphar.2023.1289227

**Published:** 2023-09-08

**Authors:** Frontiers Editorial Office

The journal retracts the 2021 article cited above.

Following publication, concerns were raised regarding the validity of the data in the article. The authors failed to provide the raw data or a satisfactory explanation during the investigation, which was conducted in accordance with Frontiers’ policies. Given the concerns, and the lack of raw data, the editors no longer have confidence in the findings presented in the article.

This retraction was approved by the Chief Editors of Frontiers in Pharmacology and the Chief Executive Editor of Frontiers. The authors agree to this retraction.

